# Measuring Core Body Temperature Using a Non-invasive, Disposable Double-Sensor During Targeted Temperature Management in Post-cardiac Arrest Patients

**DOI:** 10.3389/fmed.2021.666908

**Published:** 2021-05-05

**Authors:** David Janke, Niklas Kagelmann, Christian Storm, Martina A. Maggioni, Camilla Kienast, Hanns-Christian Gunga, Oliver Opatz

**Affiliations:** ^1^Charité–Universitätsmedizin Berlin, Corporate Member of Freie Universität Berlin, Humboldt-Universität zu Berlin and Berlin Institute of Health, Institute of Physiology, Center for Space Medicine and Extreme Environments Berlin, Berlin, Germany; ^2^Charité–Universitätsmedizin Berlin, Corporate Member of Freie Universität Berlin, Humboldt-Universität zu Berlin and Berlin Institute of Health, Department of Internal Medicine, Nephrology and Intensive Care, Berlin, Germany; ^3^Department of Biomedical Sciences for Health, University of Milan, Milan, Italy

**Keywords:** core body temperature, return of spontaneous circulation, targeted temperature management, cardiac arrest, hypothermia, intraclass correlation coefficient, heat-flux sensor

## Abstract

**Background:** Precisely measuring the core body temperature during targeted temperature management after return of spontaneous circulation is mandatory, as deviations from the recommended temperature might result in side effects such as electrolyte imbalances or infections. However, previous methods are invasive and lack easy handling. A disposable, non-invasive temperature sensor using the heat flux approach (Double Sensor), was tested against the standard method: an esophagus thermometer.

**Methods:** The sensor was placed on the forehead of adult patients (*n* = 25, M/F, median age 61 years) with return of spontaneous circulation after cardiac arrest undergoing targeted temperature management. The recorded temperatures were compared to the established measurement method of an esophageal thermometer. A paired *t*-test was performed to examine differences between methods. A Bland-Altman-Plot and the intraclass correlation coefficient were used to assess agreement and reliability. To rule out possible influence on measurements, the patients' medication was recorded as well.

**Results:** Over the span of 1 year and 3 months, data from 25 patients were recorded. The *t*-test showed no significant difference between the two measuring methods (*t* = 1.47, *p* = 0.14, *n* = 1,319). Bland-Altman results showed a mean bias of 0.02°C (95% confidence interval 0.00–0.04) and 95% limits of agreement of −1.023°C and 1.066°C. The intraclass correlation coefficient was 0.94. No skin irritation or allergic reaction was observed where the sensor was placed. In six patients the bias differed noticeably from the rest of the participants, but no sex-based or ethnicity-based differences could be identified. Influences on the measurements of the Double Sensor by drugs administered could also be ruled out.

**Conclusions:** This study could demonstrate that measuring the core body temperature with the non-invasive, disposable sensor shows excellent reliability during targeted temperature management after survived cardiac arrest. Nonetheless, clinical research concerning the implementation of the sensor in other fields of application should be supported, as well as verifying our results by a larger patient cohort to possibly improve the limits of agreement.

## Introduction

The post-resuscitation phase is critical for patients with return of spontaneous circulation (ROSC), specifically considering both the overall outcome and the quality of neurological recovery (1, 2). One of the recommended treatments after ROSC is targeted temperature management (TTM) (3) as it improves neurological outcome and survival (4–6), although there is still disagreement over the duration of the treatment (7) and the ideal temperature (3). The European Resuscitation Council Guidelines recommend limits between 32 and 36°C (3).

With decreasing core body temperature (CBT) the risk of side effects such as hypokalemia or infections might increase (8, 9) even though the quality of evidence is still moderate to low (5). Temperatures below 30°C can even increase the risk of arrhythmias (9) which makes it clear that a precise monitoring of the CBT is mandatory.

Since the hypothetical gold standard of measuring the temperature of the blood perfusing the hypothalamus is not suitable for routine CBT assessment, alternative methods have been implemented. Potential measurement sites include the pulmonary artery, the esophagus and the bladder (10).

However, a significant disadvantage of the aforementioned methods is that they are all invasive. A promising substitute are non-invasive zero-heat flux and heat flux sensors. Having first been described in the 1970s (11), these sensors use a mathematical model to calculate the CBT from temperatures measured on a perfectly insulated small skin area. The clinical value of zero-heat flux sensors has already been verified but reports cite long calibration time and a bulky sensor as inconvenient factors (12–15). Without the need for a heating element, heat flux sensors provide a quicker response time and increased wearing comfort, while delivering comparable results (16–18).

With this study a new disposable, non-invasive, heat-flux double-sensor (DS) was tested and compared it to the established method for monitoring CBT with an esophageal thermometer (OeT) during induction and maintenance of TTM as well as during rewarming in patients with ROSC after cardiac arrest.

## Materials and Methods

A prospective observational trial of a convenience sample of patients treated at the intensive care unit (ICU) at the Department of Nephrology and Medical Intensive Care of the Charité Universitätsmedizin Berlin was conducted. The sample size needed to prove excellent reliability of the DS using the Intraclass correlation coefficient (ICC) was calculated to be 16 patients (power 90%, alpha 0.05). For this, R (R Core Team, Version 4.0.3) (19) with ICC.Sample.Size (20, 21) was used with references from a similar study (22). Considering potential dropouts, 29 patients were included in the study.

Patients were recruited between November 2015 and January 2017. The in- and exclusion criteria for this study are listed in Table 1. Protocols were approved by the local ethics committee (EA4/032/16). Taking into account the underlying condition of the subjects this included waiving of informed consent. This concurs with the recommendations by the European Resuscitation Council (23). The study was conducted following the guidelines of the Declaration of Helsinki from 2013 (24). All data sets were pseudonymized and the raw data was only accessible by the author of this study. Patients or their relatives could request that the patient's data will not be included in the study.

**Table 1:** Inclusion and exclusion criteria.

**Inclusion criteria**	**Exclusion criteria**
Age ≥ 18 years	Age <18 years
Any patient after cardiac arrest with return of spontaneous circulation	
Undergoing targeted temperature management	

### Study Protocol

Following the standard operation procedures of the ICU, the TTM was initiated immediately after admitting the patient to the ward. A target temperature of 33°C was achieved with the Arctic Sun® 5000 temperature management system (BD, Heidelberg, Germany). Vital signs (including esophageal temperature), medication, procedures etc. were recorded and stored by the patient data management system Copra (Copra Systems GmbH, Berlin, Germany) every 30 min. The DS was attached to the patients' forehead before TTM was initiated.

Gloves and socks were used as countermeasures to prevent shivering during hypothermia. Pancuronium for muscle relaxation was only administered if the aforementioned arrangements were not sufficient. The target temperature was maintained for 24 h followed by a rewarming phase at a rate of 0.25°C/h. Subsequently the target temperature was held at 37°C for another 24 h to intercept any rebound fever that might occur.

**Figure 1:**
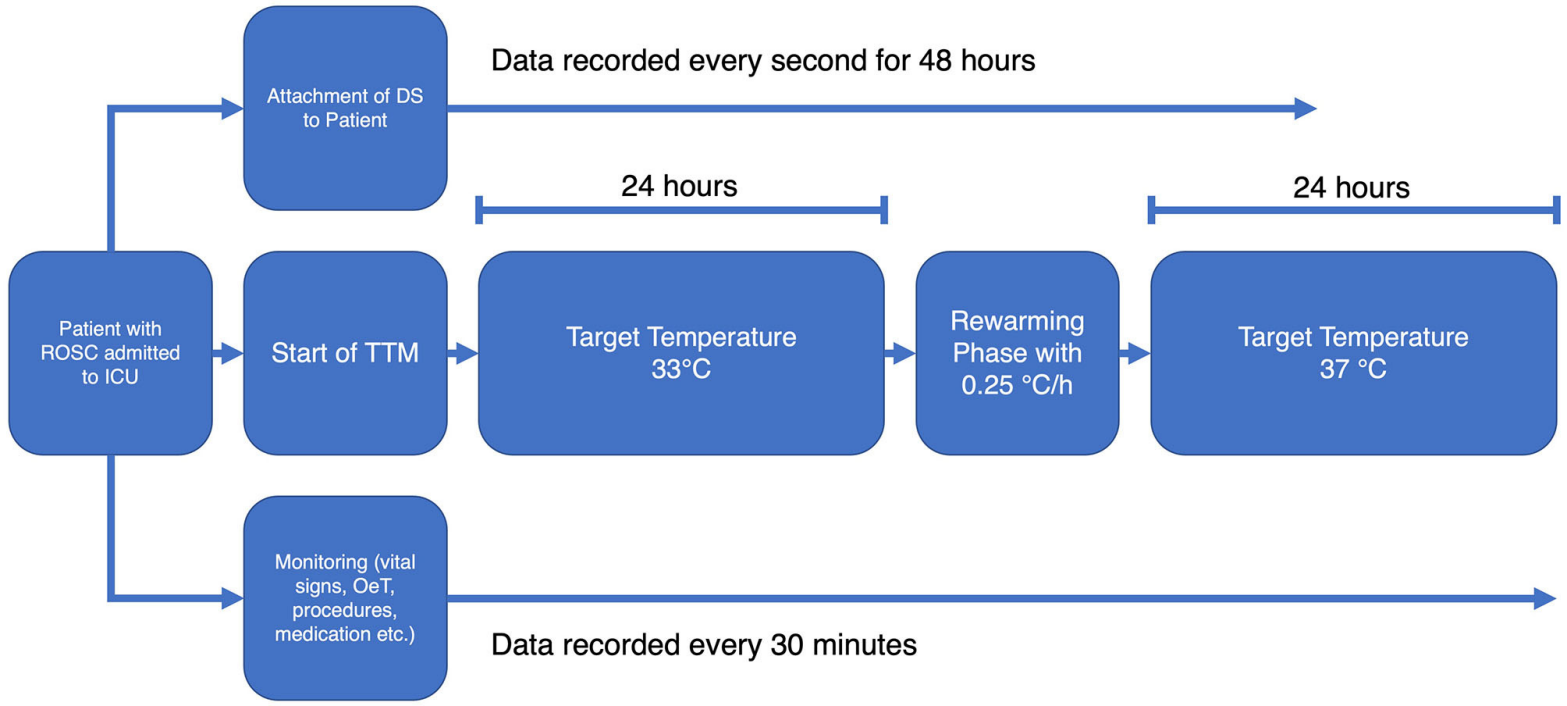
Flowchart of the study protocol.

The temperatures of the OeT and the DS were simultaneously recorded until 48 h after the start of TTM. Figure 1 shows a flowchart of the study protocol.

### Arctic Sun™ 5000 Temperature Management System

This non-invasive temperature management system monitors and controls the patient's temperature. It uses temperature-controlled water circulating through reusable pads that are wrapped around the chest and thighs of the patient. This leads to a heat exchange between the patient and the water. The patient's temperature feedback is provided by the OeT via a special connector to the control module.

### Double-Sensor and Esophageal Thermometer

**Figure 2:**
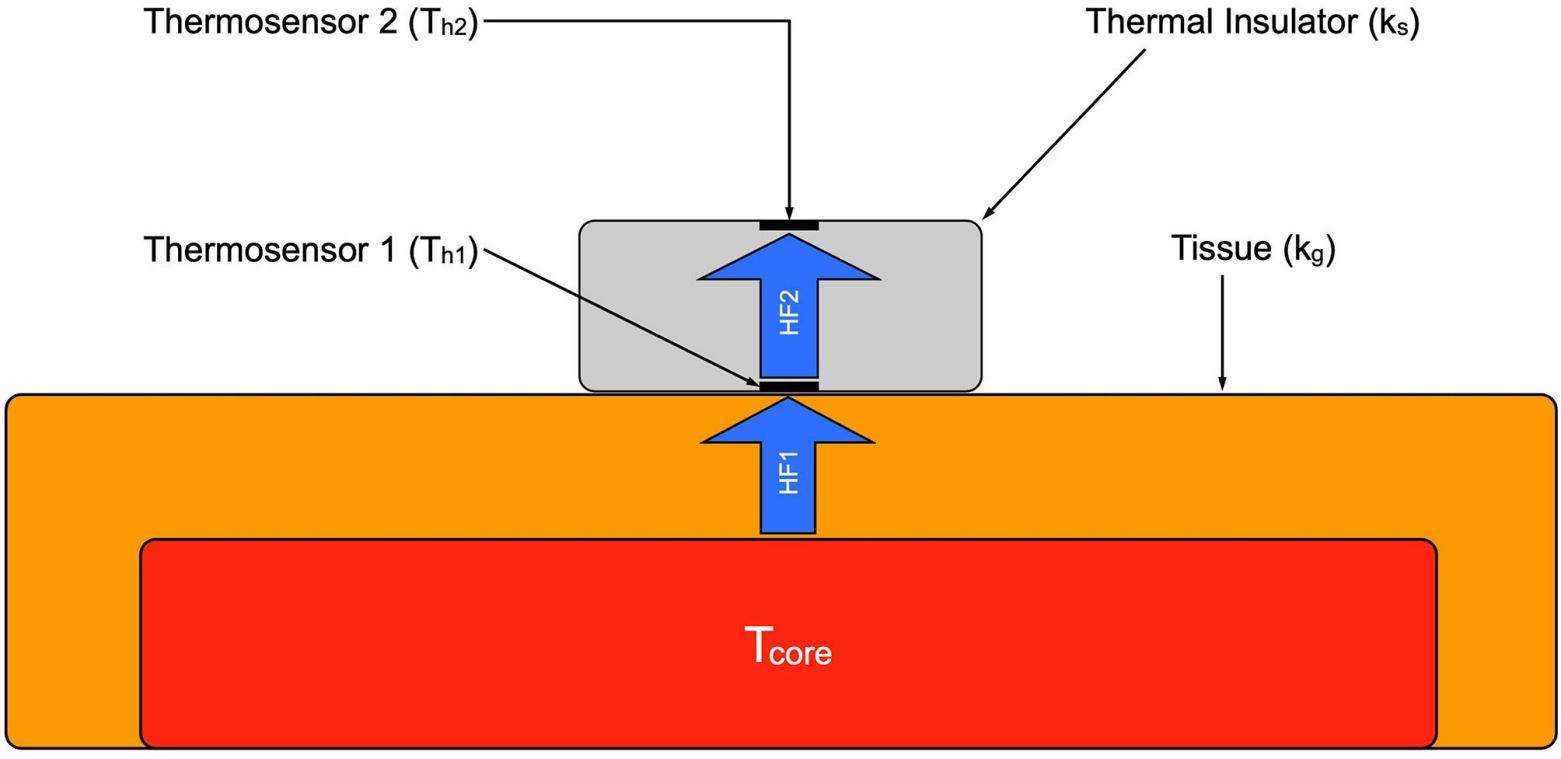
Schematic of the double sensor. Tcore, core body temperature; HF 1, heat flux from body core to the sensor; HF 2, heat flux through the sensor.

For this study the double-sensor system developed by Dräger (Draegerwerk AG & Co. KGaA, Lübeck, Germany) was used. Figure 2 shows a schematic structure. It consists of two independent temperature sensors which are separated by an insulating layer. The unit is enclosed in an isolated casing. While one sensor (T_h1_) measures the temperature of the skin, the other one (T_h2_) measures the heat flux through the sensor to the environment. The heat transfer coefficient of the insulation (K_s_) and of the human tissue (K_g_) are given. The heat flux through the insulation (HF_2_) is assumed to be equivalent to the heat flux through the skin (HF_1_). With these values it is possible to calculate the core temperature (T_core_) with the formula developed by Gunga et al. (25):

$$\begin{array}{l} {\text{T}}_{\text{core}}={\text{T}}_{\text{h}1}+{\text{K}}_{\text{s}}/{\text{K}}_{\text{g}}{\;}^{*}\;\left({\text{T}}_{\text{h}1}-{\text{T}}_{\text{h}2}\right) \end{array}$$

Once the sensor is attached to the skin continuous measurements can be conducted within a few minutes. The usage of the above-mentioned heat-flux method allows for the sensor to be considered indifferent to the ambient temperature.

Using the self-adhesive surface, the sensor was placed on the patients' forehead above the left eyebrow and afterwards connected to the data logger system (Health Lab System, Koralewski Elektronik, Hambühren, Germany). This also gives the advantage of measuring in the proximity to the organ of interest (i.e., the brain).

Temperatures for both units of the DS were recorded with a frequency of 1/s and stored with a timestamp to the data logger. To ensure the internal validity of the recording and to rule out any influence on the measurement, environmental data were recorded, such as air pressure, ambient temperature and humidity. Data from the data logger were exported using SpaceBit Heally HLCC (Koralewski Elektronik, Hambuhren, Germany).

The esophagus thermometer in use was the Mon-a-Therm™ (Mallinckrodt Inc., St Louis, MO, USA) and was placed in the distal third of the esophagus at approximately 30 cm lip level. Given the proximity to the left atrium a good estimate of the CBT can be obtained from there (26). The preset recording frequency for the OeT was every 30 min, which unfortunately could not be changed beforehand without intensive reprogramming of the patient data management system.

### Sedation, Analgesia and Other Vasoactive Agents

Analgosedation was achieved using Midazolam, Ketamine or Propofol in combination with Sufentanil. Isoflurane in the Anesthetic Conserving Device (AnaConDa, Sedana Medical AB, Sweden) combined with Remifentanil was preferably used whenever feasible mainly because of the short half-life with low risk of accumulation and rapid reawakening. To account for the effect which most inhalative and intravenous agents for sedation have on vasomotion (27) and consequently on the DS measurements, dosages and flow rate of drugs administered were recorded.

### Data Analysis

The data sets were analyzed using MS Excel (Version 16.16.20) as well as IBM SPSS (Version 26.0.0). DS derived core temperature was calculated from temperatures Th_1_ and Th_2_ with the formula mentioned above. A two-sided *p*-value of < 0.05 was considered statistically significant. Continuous data are reported as means and standard deviation (SD). Artifacts were defined as difference > 2 SD. After proving the normal distribution of the data sets, a *t*-test for paired samples was used to examine the difference between methods. Furthermore, mean differences and standard deviation were calculated and used for a Bland-Altman Plot (28). The acceptable limits of agreement (LoA) were defined *a priori* as ± 0.5°C. These limits have been used in previous studies (12, 13, 16–18) and correspond to the usual magnitude of the human circadian temperature variation (29, 30). The intraclass correlation coefficient (ICC) (31, 32) was additionally used to evaluate the agreement and correlation between the OeT and the DS on the CBT. ICC estimates and their 95% confident intervals (CI) were calculated based on a single-rating (*k* = 2), consistency-agreement and a 2-way mixed effects model.

For the classification of the ICC Cicchetti's (33) definition is commonly used with an ICC < 0.4 indicating a poor, between 0.4 and 0.59 a moderate, between 0.6 and 0.74 a good and > 0.75 an excellent level of reliability.

## Results

Twenty nine patients were initially included in the study. In the process of data analysis four patients were excluded because of data storage errors due to a malfunction of the data logger's battery. This was resolved by switching to lithium-ion batteries. Epidemiologic data for the remaining patients are listed in Table 2.

**Table 2:** Epidemiologic data.

*N*	25	
Sex, *n* (%)	Female	5 (20%)
	Male	20 (80%)
Age in years, mean (SD)	60 (12)	
BMI in kg/m^2^, mean (SD)	27 (3.6)	
Length of stay in days, mean (SD)	11.49 (8.85)	
Initial rhythm, *n* (%)	VF	12 (48%)
	Asystole	6 (24%)
	PEA	7 (28%)
Adrenaline administered during CPR in mg, mean (SD)	4 (5)	
Time to ROSC in minutes, mean (SD)	22 (18)	
Out-of-hospital cardiac arrest, *n* (%)	21 (84%)	
Collapse witnessed, *n* (%)	22 (88%)	
Bystander CPR, *n* (%)	18 (72%)	
Admission diagnosis, *n* (%)	Acute myocardial infarction	12 (48%)
	Cardiac arrhythmia	1 (4%)
	Hyperkalemia	2 (8%)
	Respiratory insufficiency	7 (28%)
	Cardiogenic shock	1 (4%)
	Electrical accident	1 (4%)
	Other	1 (4%)
SAPSII at admission, mean (SD)	62 (17)	
Discharged from hospital, *n* (%)	10 (40%)	

*BMI, body mass index; VF, ventricular fibrillation; PEA, pulseless electrical activity; CPR, cardiopulmonary resuscitation; ROSC, return of spontaneous circulation; SAPSII, simplified acute physiology score II*.

**Figure 3:**
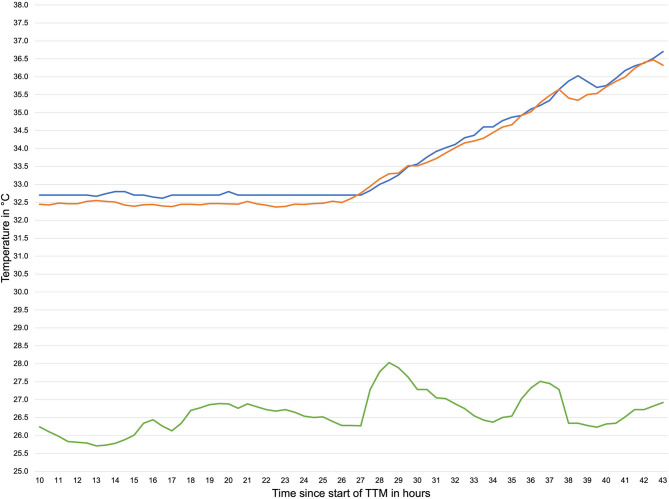
Exemplary temperature profile during TTM. orange line: temperature measured by double sensor, blue line: temperature measured by esophageal thermometer, green line: ambient temperature.

In total 2,695,806 temperature data samples were recorded with the DS and 15,084 with the OeT. After adjusting to the different sample rates of the DS and the OeT and the removal of artifacts (95 temperature pairs; 6.7%) this led to 1,319 time-paired temperature samples. The mean temperature for the DS was 34.11°C (SD 1.63°C) ranging from 29.3 to 38.03°C and 34.13°C (SD 1.42°C) ranging from 28.76 to 37.26°C for the OeT. Seventy one percent of the data recorded with the DS were in between ± 0.5°C of the temperatures recorded with the OeT. Mean ambient temperature was 25.22°C (SD 1.48°C). Figure 3 shows an exemplary temperature profile of one patient.

**Figure 4:**
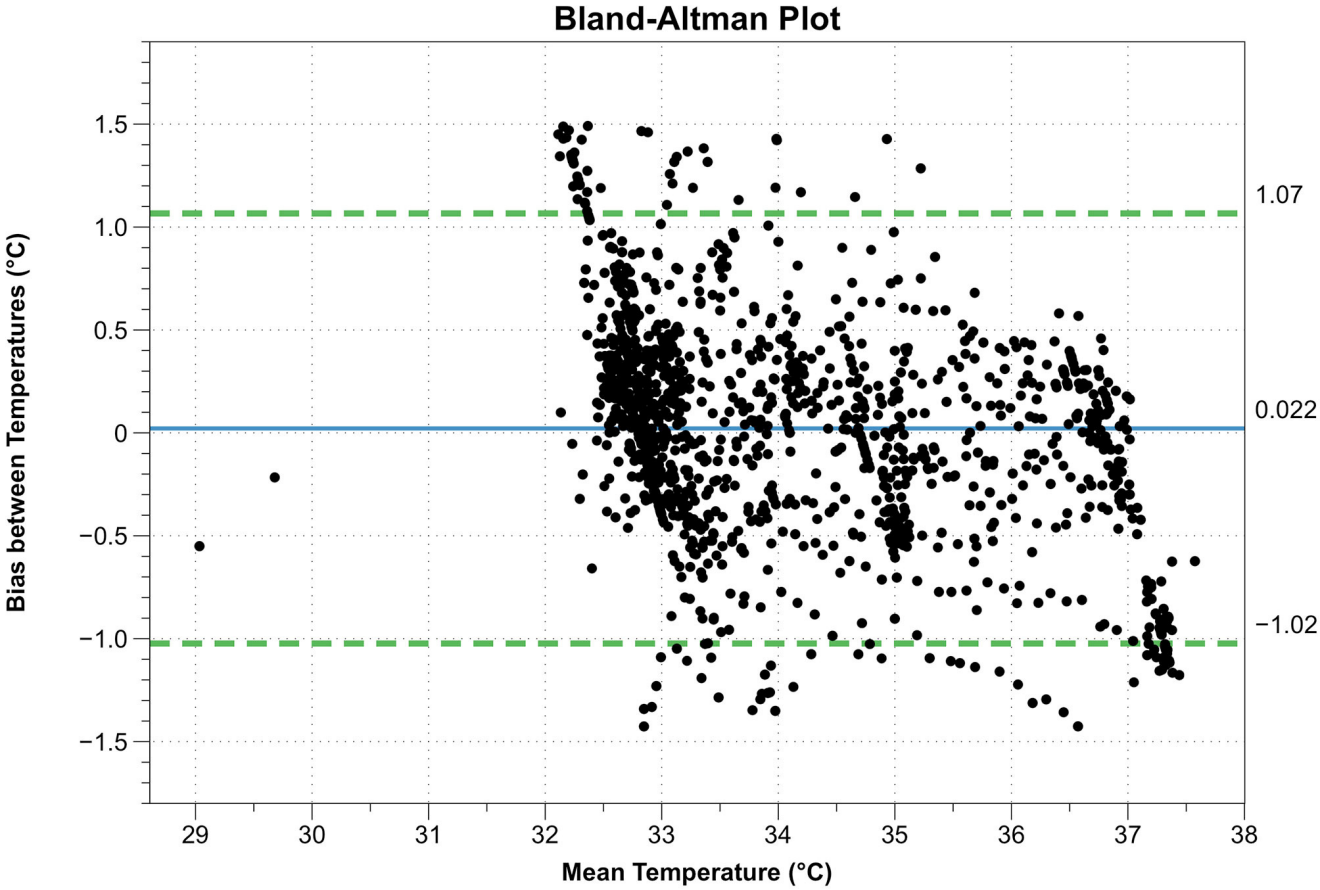
Bland-Altman plot. X-axis: mean temperature between esophagus thermometer and double sensor, Y-axis: difference between esophagus thermometer and double sensor, continuous blue line: mean difference between esophagus thermometer and double sensor, dashed green lines: limits of agreement (= mean difference ± 1.96 standard deviations).

The calculated data are shown in Table 3. The paired sample *t*-test showed no difference between the means of the two sensors (*t* = 1.47, *p* = 0.14, *n* = 1,319). The Bland-Altman plot is shown in Figure 4.

**Table 3:** Agreements between double-sensor and esophageal temperature.

**Double sensor vs. esophageal temperatures**		**95% CI**	***P*-value**
Mean bias in °C	0.02	0.0–0.04	0.14
SD in °C	0.53		
95% LoA in °C (Bias ± 1.96*SD)	−1.023; +1.066	Lower LoA: −1.025– −1.022 Upper LoA: 1.065–1.068	
ICC (95% CI)	0.94	0.93–0.95	<0.001

*CI, confidence interval; SD, standard deviation; ICC, intraclass correlation coefficient; LoA, limits of agreement*.

In one case the bias suddenly increased from −0.03 to −1.01°C within 17 h and in a second case from −0.23 to −1.43°C within 14 h. In both cases the DS reported an increase in temperature ~3 to 4 h before the OeT. Four more cases with a noticeable baseline bias were identified and are shown with the other two cases in Table 4.

**Table 4:** Patients with a noticeable difference in the bias between temperatures measured between esophageal temperatures and the double sensor.

**Patient ID**	**Sex**	**Mean bias (SD) in ^**°**^C**	**Minimum bias in ^**°**^C**	**Maximum bias in ^**°**^C**	**BMI in kg/m^**2**^**
7	m	0.88 (0.47)	0.21	1.49	21.4
10	f	−0.53 (0.88)	−0.09	1.42	27.5
14	m	−0.4 (0.34)	0.00	−1.01	29.1
18	m	−0.8 (0.37)	0.02	−1.43	26.1
23	m	0.68 (0.5)	0.02	1.47	35.1
25	m	0.87 (0.12)	0.66	1.19	29.2

*m, male; f, female; SD, standard deviation; BMI, body mass index*.

The temperatures for obese patients with a Body Mass Index (BMI) ≥ 30 kg/m^2^ (*n* = 3) were similar to the whole group except for one case. No sex-based or ethnicity-based differences were detected.

## Discussion

For this clinical study the capabilities of a new disposable heat flux sensor were tested on patients undergoing TTM after cardiac arrest and compared to the established method of an OeT. This setting requires the ability to measure temperatures both precisely and over a relatively wide range of temperatures (~6°C).

To date, the gold standard for measuring CBT is the temperature in the pulmonary artery (34, 35). However, Stone et al. (10) observed that during cardiac arrest or deep hypothermia the pulmonary artery's temperature does not always correlate with the brain's temperature. The esophageal temperature, however, corresponded closely and combines accuracy, response time and invasiveness appropriately. Nonetheless, the OeT is not the ideal tool: the correct placement of the probe in the distal esophagus is necessary to acquire precise data and to prevent inspired gases from distorting the measurements (36). It is also not suitable for use during esophageal interventions and some head and neck surgical procedures. Furthermore, this method causes discomfort in awake patients, so the thermometer is usually removed once the patients regain consciousness and is replaced by less precise (e.g., bladder temperature, rectal temperature) (10, 34) or even discontinuous methods (e.g., spot checking with an infrared thermometer, axillary thermometer). In contrast, the DS provides easy handling and is much more tolerable for the patient. Previously described skin irritations (12, 16, 17) were not observed in this study. Neither did this study show the often cited (17, 37) extensive calibration time. Two patients' data sets were identified, which allowed for a direct comparison of the two thermometers' initial parallel recording performance: after 2 min (patient 1, bias of 0.25°C) and after 3 min (patient 2, bias of 0.36°C) the first data pairs were recorded. A higher frequency of esophageal temperature measurements is necessary to generate more comparable data pairs. But from these two examples it can be concluded that the calibration time amounts to <3 min.

The statistical analysis of a comparison between two sensors requires the consideration of two decisive details:

- First, not only correlation, but also agreement must be examined. Most studies use Pearson's r to measure correlation omitting their agreement. In order to assess the reproducibility between the different methods the ICC was used which also quantifies the agreement. In contrast to other correlation coefficients, it also takes the systematic error into account, which makes it a great alternative for showing agreement between two methods (38). According to the classifications described earlier the calculated ICC of 0.94 indicates excellent reliability for determining the CBT for the DS in this study.
- Second, precision and accuracy must be ensured. The OeT is widely considered to be of equivalent quality to the existing gold standard (10) which attests both precision and accuracy. This study showed a high level of agreement between the esophagus sensor and the DS, extending these attributes to the DS as well. Taking these analytical details into account the DS can be considered a reliable alternative.

A limitation to this study was the relatively small paired-sample size in comparison to the overall number of collected samples for each method. One of the reasons for this discrepancy was the low data collection frequency of the OeT which could not be changed beforehand. Another limiting factor were interruptions of the data collection through the Copra system for patients who underwent percutaneous coronary intervention as part of the post-arrest treatment (3) after their admission to the ICU. The DS however was still attached to the patients' forehead and continued recording. Lastly the above-mentioned technical problems of the data logger disrupted measurements in a few cases before the end of the 48-h period. These disruptions occurred even after the batteries had been replaced and after a software bug had been ruled out, thus making accidental manipulation of the data logger through nursing or physician staff (e.g., while positioning or examining the patient) the most likely cause.

Another limitation is the discrepancy between a low bias and wide limits of agreement. Our percentage of values diverging 0.5°C from the mean bias is comparable to results by Eshraghi et al. (13) (71 vs. 78%), suggesting homogeneity for most of the recordings. Six patients were identified with a larger bias than the rest seen in Table 4. One patient had a BMI > 35 kg/m^2^. Since the sensor works best on skin with low subcutaneous fat tissue (39) and few large veins (40), the patients' obesity might account for the high bias in this case. Two other patients' data sets showed a gradually increasing offset between the two sensors' measurements after 12 (Patient ID 7) or 24 h (Patient ID 18) from the start of the recording. The DS registered higher temperatures approximately 4 h before the esophagus sensor measured the same increased values. Dosages and flow rates in agents influencing vasomotion were constant for both patients during this time. The remaining three patients showed a constant larger bias while following the temperature trend of the OeT. Possible explanations for all patients that were ruled out were prone positioning of the patient, unintentional covering or removal of the DS as well as changes in the ambient temperature. Additionally, Mazgaoker et al. (41) demonstrated that the DS measurements were not affected by changes in the environmental temperature. Another explanation is given by Opatz et al. (42), who found a non-linear relationship between sensor sites to measure the CBT. The more remote the sensor position from the organ of interest, the greater the effect of non-linearity. This means that the increase in temperature as measured by the DS is not linear to the temperature in the esophagus as it is further away from the brain (the organ of interest). The authors state that the time lag between sensor positions is not constant but individual for each patient. Further study is needed to evaluate contributory factors since this phenomenon only occurred in two of our patients and after a certain amount of time.

Another possible factor influencing our results is the medication regimen of patients in the ICU. General anesthetics and opioids (e.g., Propofol, Dexmedetomidine, Isoflurane, Clonidine, Fentanyl) decrease the cold-response threshold and thereby the vasoconstriction threshold (27, 43), which could interfere with the measurements. However, Ikeda et al. (44) showed that anesthesia had almost no effect on the core-to-skin temperature gradient.

To our knowledge this is the only study testing the Draeger DS in such a setting to date. Zeiner et al. (12) had a comparable setting but used a prototype zero-heat flux sensor. The results were similar with a bias of −0.12°C but with smaller limits of agreement. Other studies were mostly set in an operational setting (15, 17, 18, 40, 42) or compared the heat flux principle to nasopharyngeal (14), pulmonary arterial (13, 14), or vesical (42) temperature. Even though most of the studies report the use of a zero-heat flux sensor with a heating element or a sensor from a different manufacturer the results are similar. A recently published review by Conway et al. (45) on the use of the 3M™ heat flow sensor supports this statement.

Building on the findings of our study we recommend three further steps:

1. Verifying our results by a larger patient cohort to possibly improve the limits of agreement.
2. Modification of the recording system of the OeT in order to generate more data pairs per patient and time unit.
3. Additionally, further possible applications for this type of sensor need to be explored. The lack of an omnipotent temperature sensor and a large variety of application settings call for the use of the DS as a non-invasive alternative to the established methods.

So far, however, the DS technology has not yet been established in clinical practice. The possible reasons for this are diverse, as Wartzek et al. (46) show in their review. As a way of implementing this method into clinical standards, the use as a complementary secondary monitoring site to evaluate other temperature measurements is suggested. It could also be incorporated into other monitoring devices such as electroencephalography, electrocardiogram, SpO_2_ etc. In conclusion this study showed that the DS is a reliable and non-invasive tool to measure the CBT in patients during TTM after cardiac arrest and ROSC. Further clinical research concerning the implementation of the sensor in other fields of application should be supported.

## Data Availability Statement

The raw data supporting the conclusions of this article will be made available by the authors, without undue reservation.

## Ethics Statement

This study was approved by Ethics committee Charité Universitätsmedizin Berlin (EA4/032/16). Considering the underlying condition of patients, waiving of informed consent, when necessary, was accepted. This concurs with the recommendations by the European Resuscitation Council (23).

## Author Contributions

OO: conceptualization and project administration. OO, CS, and MM: methodology. CS: software, investigation, and resources. DJ and CS: validation. DJ: formal analysis, visualization, and writing—original draft. OO, NK, H-CG, MM, and CK: writing—review and editing. OO and CS: supervision. All authors contributed to the article and approved the submitted version.

## Conflict of Interest

The authors declare that the research was conducted in the absence of any commercial or financial relationships that could be construed as a potential conflict of interest.

## References

[1] Carr BG Goyal M Band RA Gaieski DF Abella BS Merchant RM. A national analysis of the relationship between hospital factors and post-cardiac arrest mortality. Intensive Care Med. (2009) 35:505–11. 10.1007/s00134-008-1335-x 18936907

[2] Spaite DW Bobrow BJ Stolz U Berg RA Sanders AB Kern KB. Statewide regionalization of postarrest care for out-of-hospital cardiac arrest: association with survival and neurologic outcome. Ann Emerg Med. (2014) 64:496–506.e1. 10.1016/j.annemergmed.2014.05.028 25064741

[3] Nolan JP Soar J Cariou A Cronberg T Moulaert VRM Deakin CD. European Resuscitation Council and European Society of Intensive Care Medicine Guidelines for Post-resuscitation Care 2015: Section 5 of the European Resuscitation Council Guidelines for Resuscitation 2015. Resuscitation. (2015) 95:202–22. 10.1016/j.resuscitation.2015.07.018 26477702

[4] Testori C Sterz F Behringer W Haugk M Uray T Zeiner A. Mild therapeutic hypothermia is associated with favourable outcome in patients after cardiac arrest with non-shockable rhythms. Resuscitation. (2011) 82:1162–7. 10.1016/j.resuscitation.2011.05.022 21705132

[5] Arrich J Holzer M Havel C Müllner M Herkner H. Hypothermia for neuroprotection in adults after cardiopulmonary resuscitation. Cochrane Database Syst Rev. (2016) 2:CD004128. 10.1002/14651858.CD004128.pub4 26878327 PMC6516972

[6] Hypothermia after Cardiac Arrest Study Group. Mild therapeutic hypothermia to improve the neurologic outcome after cardiac arrest. N Engl J Med. (2002) 346:549–56. 10.1056/NEJMoa012689 11856793

[7] Kirkegaard H Søreide E de Haas I Pettilä V Taccone FS Arus U. Targeted temperature management for 48 vs 24 hours and neurologic outcome after out-of-hospital cardiac arrest: a randomized clinical trial. JAMA. (2017) 318:341–50. 10.1001/jama.2017.8978 28742911 PMC5541324

[8] Silverman MG Scirica BM. Cardiac arrest and therapeutic hypothermia. Trends Cardiovasc Med. (2016) 26:337–44. 10.1016/j.tcm.2015.10.002 26603661

[9] Polderman KH Herold I. Therapeutic hypothermia and controlled normothermia in the intensive care unit: practical considerations, side effects, and cooling methods. Crit Care Med. (2009) 37:1101–20. 10.1097/CCM.0b013e3181962ad5 19237924

[10] Stone JG Young WL Smith CR Solomon RA Wald A Ostapkovich N. Do standard monitoring sites reflect true brain temperature when profound hypothermia is rapidly induced and reversed? Anesthesiology. (1995) 82:344–51. 10.1097/00000542-199502000-00004 7856892

[11] Fox RH Solman AJ. A new technique for monitoring the deep body temperature in man from the intact skin surface. J Physiol. (1971) 212:8P𢀓10P. 5548025

[12] Zeiner A Klewer J Sterz F Haugk M Krizanac D Testori C. Non-invasive continuous cerebral temperature monitoring in patients treated with mild therapeutic hypothermia: an observational pilot study. Resuscitation. (2010) 81:861–6. 10.1016/j.resuscitation.2010.03.018 20398992

[13] Eshraghi Y Nasr V Parra-Sanchez I Van Duren A Botham M Santoscoy T. An evaluation of a zero-heat-flux cutaneous thermometer in cardiac surgical patients. Anesth Analg. (2014) 119:543–9. 10.1213/ANE.0000000000000319 25045862

[14] Sastre JA Pascual MJ López T. Evaluation of the novel non-invasive zero-heat-flux Tcore™ thermometer in cardiac surgical patients. J Clin Monit Comput. (2019) 33:165–72. 10.1007/s10877-018-0143-2 29667096

[15] Boisson M Alaux A Kerforne T Mimoz O Debaene B Dahyot-Fizelier C. Intra-operative cutaneous temperature monitoring with zero-heat-flux technique (3M SpotOn) in comparison with oesophageal and arterial temperature: a prospective observational study. Eur J Anaesthesiol. (2018) 35:825–30. 10.1097/EJA.0000000000000822 29708906

[16] Gunga HC Werner A Stahn A Steinach M Schlabs T Koralewski E. The Double Sensor-A non-invasive device to continuously monitor core temperature in humans on earth and in space. Respir Physiol Neurobiol. (2009) 169 Suppl 1:S63–8. 10.1016/j.resp.2009.04.005 19428314

[17] Kimberger O Thell R Schuh M Koch J Sessler DI Kurz A. Accuracy and precision of a novel non-invasive core thermometer. Br J Anaesth. (2009) 103:226–31. 10.1093/bja/aep134 19482858

[18] Kimberger O Saager L Egan C Sanchez IP Dizili S Koch J. The accuracy of a disposable noninvasive core thermometer. Can J Anaesth. (2013) 60:1190–6. 10.1007/s12630-013-0047-z 24214518

[19] R Core Team. R: A Language and Environment for Statistical Computing. 3.6.3 ed. Vienna: R Foundation for Statistical Computing (2020).

[20] Rathbone A Shaw S Kumbhare D. ICC.Sample.Size: Calculation of Sample Size and Power for ICC. R Package Version 1.0. (2015). Available online at: https://CRAN.R-project.org/package=ICC.Sample.Size

[21] Zou GY. Sample size formulas for estimating intraclass correlation coefficients with precision and assurance. Stat Med. (2012) 31:3972–81. 10.1002/sim.5466 22764084

[22] Kollmann Camaiora A Brogly N Alsina E de Celis I Huercio I Gilsanz F. Validation of the zero-heat-flux thermometer (SpotOn®) in major gynecological surgery to monitor intraoperative core temperature: a comparative study with esophageal core temperature. Minerva Anestesiol. (2019) 85:351–7. 10.23736/S0375-9393.18.12188-2 29945430

[23] Bossaert LL Perkins GD Askitopoulou H Raffay VI Greif R Haywood KL. European Resuscitation Council Guidelines for Resuscitation 2015: Section 11. The ethics of resuscitation and end-of-life decisions. Resuscitation. (2015) 95:302–11. 10.1016/j.resuscitation.2015.07.033 26477419

[24] Association WM. World medical association declaration of helsinki: ethical principles for medical research involving human subjects. JAMA. (2013) 310:2191–4. 10.1001/jama.2013.281053 24141714

[25] Gunga H-C Sandsund M Reinertsen RE Sattler F Koch J. A non-invasive device to continuously determine heat strain in humans. J Therm Biol. (2008) 33:297–307. 10.1016/j.jtherbio.2008.03.004

[26] Pasquier M Paal P Kosinski S Brown D Podsiadlo P Darocha T. Esophageal temperature measurement. N Engl J Med. (2020) 383:e93. 10.1056/NEJMvcm1900481 33053286

[27] Sessler DI. Perioperative thermoregulation and heat balance. Lancet. (2016) 387:2655–64. 10.1016/S0140-6736(15)00981-2 26775126

[28] Bland JM Altman DG. Statistical methods for assessing agreement between two methods of clinical measurement. Lancet. (1986) 1:307–10. 2868172

[29] Sessler DI Lee KA McGuire J. Isoflurane anesthesia and circadian temperature cycles in humans. Anesthesiology. (1991) 75:985–9. 10.1097/00000542-199112000-00010 1741520

[30] Tayefeh F Plattner O Sessler DI Ikeda T Marder D. Circadian changes in the sweating-to-vasoconstriction interthreshold range. Pflugers Arch. (1998) 435:402–6. 10.1007/s004240050530 9426297

[31] Bartko JJ. The intraclass correlation coefficient as a measure of reliability. Psychol Rep. (1966) 19:3–11. 10.2466/pr0.1966.19.1.3 5942109

[32] Shrout PE Fleiss JL. Intraclass correlations: uses in assessing rater reliability. Psychol Bull. (1979) 86:420–8. 10.1037//0033-2909.86.2.420 18839484

[33] Cicchetti DV. Guidelines, criteria and rules of thumb for evaluating normed and standardized assessment instruments in psychology. Psychol Assess. (1994) 6:284–90. 10.1037/1040-3590.6.4.284

[34] Akata T Setoguchi H Shirozu K Yoshino J. Reliability of temperatures measured at standard monitoring sites as an index of brain temperature during deep hypothermic cardiopulmonary bypass conducted for thoracic aortic reconstruction. J Thorac Cardiovasc Surg. (2007) 133:1559–65. 10.1016/j.jtcvs.2006.11.031 17532957

[35] Peron P. The choice of the method for body temperature measurement in intensive care patients: a literature review. Prof Inferm. (2010) 63:99–105. 20943098

[36] Imrie MM Hall GM. Body temperature and anaesthesia. Br J Anaesth. (1990) 64:346–54. 10.1093/bja/64.3.346 2183863

[37] Yamakage M Namiki A. Deep temperature monitoring using a zero-heat-flow method. J Anesth. (2003) 17:108–15. 10.1007/s005400300026 12903922

[38] van Stralen KJ Jager KJ Zoccali C Dekker FW. Agreement between methods. Kidney Int. (2008) 74:1116–20. 10.1038/ki.2008.306 18596728

[39] Togawa T. Non-invasive deep body temperature measurement. In: Rolfe P editor. Non-invasive physiological measurements. London; New York: Academic Press, London (1979). p. 261–77.

[40] Teunissen LPJ Klewer J de Haan A de Koning JJ Daanen HAM. Non-invasive continuous core temperature measurement by zero heat flux. Physiol Meas. (2011) 32:559–70. 10.1088/0967-3334/32/5/005 21444968

[41] Savyon Mazgaoker IK Ran Yanovich Yuval Heled Yoram Epstein. Measuring core body temperature with a non-invasive sensor. J Therm Biol. (2017) 66:17–20. 10.1016/j.jtherbio.2017.03.007 28477905

[42] Opatz O Trippel T Lochner A Werner A Stahn A Steinach M. Temporal and spatial dispersion of human body temperature during deep hypothermia. Br J Anaesth. (2013) 111:768–75. 10.1093/bja/aet217 23801744

[43] Sessler DI. Temperature monitoring and perioperative thermoregulation. Anesthesiology. (2008) 109:318–38. 10.1097/ALN.0b013e31817f6d76 18648241 PMC2614355

[44] Ikeda T Sessler DI Marder D Xiong J. Influence of thermoregulatory vasomotion and ambient temperature variation on the accuracy of core-temperature estimates by cutaneous liquid-crystal thermometers. Anesthesiology. (1997) 86:603–12. 10.1097/00000542-199703000-00012 9066326

[45] Conway A Bittner M Phan D Chang K Kamboj N Tipton E. Accuracy and precision of zero-heat-flux temperature measurements with the 3M Bair Hugger Temperature Monitoring System: a systematic review and meta-analysis. J Clin Monit Comput. (2020). 10.1007/s10877-020-00543-6 32488679

[46] Wartzek T Mühlsteff J Imhoff M. Temperature measurement. Biomed Tech. (2011) 56:241–57. 10.1515/BMT.2011.108 21988157

